# Characterization and Behaviour of Silica Engineered Nanocontainers in Low and High Ionic Strength Media

**DOI:** 10.3390/nano13111738

**Published:** 2023-05-26

**Authors:** Violeta Ferreira, Joana Figueiredo, Roberto Martins, Alesia Sushkova, Frederico Maia, Ricardo Calado, João Tedim, Susana Loureiro

**Affiliations:** 1 CESAM—Centre for Environmental and Marine Studies & Department of Biology, University of Aveiro, 3810-193 Aveiro, Portugal; violeta@ua.pt (V.F.); jrmf@ua.pt (J.F.); roberto@ua.pt (R.M.); rjcalado@ua.pt (R.C.); 2 CICECO—Aveiro Institute of Materials & Department of Materials and Ceramic Engineering, University of Aveiro, 3810-193 Aveiro, Portugal; alesia@ua.pt (A.S.); joao.tedim@ua.pt (J.T.); 3 Smallmatek—Small Materials and Technologies, Lda., Rua Canhas, 3810-075 Aveiro, Portugal; frederico.maia@smallmatek.pt

**Keywords:** agglomeration, aggregation, antifouling, DCOIT, mesoporous silica, marine, stability

## Abstract

Mesoporous silica engineered nanomaterials are of interest to the industry due to their drug-carrier ability. Advances in coating technology include using mesoporous silica nanocontainers (SiNC) loaded with organic molecules as additives in protective coatings. The SiNC loaded with the biocide 4,5-dichloro-2-octyl-4-isothiazolin-3-one (DCOIT), i.e., SiNC-DCOIT, is proposed as an additive for antifouling marine paints. As the instability of nanomaterials in ionic-rich media has been reported and related to shifting key properties and its environmental fate, this study aims at understanding the behaviour of SiNC and SiNC-DCOIT in aqueous media with distinct ionic strengths. Both nanomaterials were dispersed in (i) low- (ultrapure water—UP) and (ii) high- ionic strength media—artificial seawater (ASW) and f/2 medium enriched in ASW (f/2 medium). The morphology, size and zeta potential (ζP) of both engineering nanomaterials were evaluated at different timepoints and concentrations. Results showed that both nanomaterials were unstable in aqueous suspensions, with the initial ζP values in UP below −30 mV and the particle size varying from 148 to 235 nm and 153 to 173 nm for SiNC and SiNC-DCOIT, respectively. In UP, aggregation occurs over time, regardless of the concentration. Additionally, the formation of larger complexes was associated with modifications in the ζP values towards the threshold of stable nanoparticles. In ASW, SiNC and SiNC-DCOIT formed aggregates (<300 nm) independently of the time or concentration, while larger and heterogeneous nanostructures (>300 nm) were detected in the f/2 medium. The pattern of aggregation detected may increase engineering nanomaterial sedimentation rates and enhance the risks towards dwelling organisms.

## 1. Introduction

In Europe, more than one million tons of silica engineering nanomaterials (ENMs) were traded in 2022 [1]. Thus, increasing amounts of silica-based ENMs are expected in natural compartments through the release of urban and industrial mismanaged sewage effluents [2].

Several types of silica ENMs (e.g., fumed silicas, silica sols, mesoporous silica, etc.) can be used in different industrial applications [3,4]. In particular, mesoporous silica nanocontainers (SiNCs) gained interest in generating drug-delivery systems and stimuli-responsive nanocarriers [5,6] due to their hollow structure, high surface area, tunable pore size, biocompatibility and low-cost synthesis [3]. The inclusion of SiNC additives in marine protective coatings has been highlighted due to the successful encapsulation and controlled release of antifouling biocides [7,8,9] and anticorrosion agents [10,11,12]. These ENMs showed effective biocidal activity and a reduced environmental footprint compared to traditional marine coatings by controlling chemical release over time [7,13,14,15]. However, paint particles are often released into the water column during paint application, boat maintenance activities or due to uncontrolled leaching [16], which may negatively impact aquatic species [17,18,19].

Given their high surface area, ENMs may experience homo- and hetero-aggregation in natural environments and undergo other physicochemical processes, such as sedimentation, adsorption or coating. Those processes are known to modulate the fate and bioavailability of ENM [20,21] and are influenced by abiotic factors, such as ionic strength or the presence of dissolved organic material [22,23,24,25]. Few studies have addressed the influence of those factors on the stability of mesoporous silica nanocontainers [12,26,27] comparatively to what is described for other metal nanomaterials [28,29,30].

Despite the progress in understanding the interactions of ENMs in environmental matrices [31], the behaviour of ENMs at their end-life stages when reaching marine areas remains limited [32,33]. Insights into the transformations experienced by ENMs under those conditions will support the generation of safer nano-based products [34].

The characterization of silicon nanoparticles, structurally distinct from SiNC, in seawater, confirmed the formation of aggregates of different sizes with implications for ENM ecotoxicity [35,36,37,38] and transport in seawater [39]. Little is known about the colloidal stability of mesoporous silica ENMs once released into the aquatic compartment [26,40], and no clear pattern linking toxicity with particle size has been established yet. For instance, Bondarenko et al. [40] performed an ecotoxicology survey testing mesoporous silica ENMs in freshwater and marine species. The authors reported no toxicity (EC_50_ > 100 mg/L) in all models except for freshwater microalgae (EC_50_ = 83.6 mg/L). In parallel, the hydrodynamic size of the ENM was estimated in different test media. The silica nanoparticles detected in freshwater (575 ± 65 nm) were smaller than what was found in the saline medium (1101 ± 76 nm), which suggests that particle size may have implications for the toxicological effects of this ENM. On the other hand, Figueredo et al. [26], who tested mesoporous silica particles with a hydrodynamic size between 180 nm and 708 nm, observed that the sensitivity of marine organisms to SiNC was species-specific and independent of particle size.

In the present study, the ENMs (1) hollow silica nanocapsules (SiNC) and (2) SiNC loaded with the antifouling biocide 4,5-dichloro-2-octyl-4-isothiazolin-3-one (SiNC-DCOIT), two forms of mesoporous silica nanomaterials, were characterized in terms of stability in media of different ionic strengths and concentrations, through time. The results gather data on the sizing and electric charge of these ENMs in standardized test media used in ecotoxicological surveys: artificial seawater and f/2-enriched artificial seawater medium, plus ultrapure water. The dataset will help unveil the transformations experienced by these ENMs when reaching the ocean.

## 2. Materials and Methods

### 2.1. Materials

Acetonitrile and methanol (HPLC grade) were purchased from Fisher Scientific (Hampton, NH, USA). The SEA-NINE™ 211N (30% of DCOIT in xylene) was obtained from Rohm and Haas (Philadelphia, PA, USA). Tetraethoxysilane (TEOS, 99.9%), cetyltrimethylammonium bromide (CTAB, >98%) was supplied by Sigma-Aldrich (St. Louis, MO, USA). Pro-Reef salt was purchased from Tropic Marin^®^ (Wartenberg, Germany). Analytical grade xylene was provided by Labscan (Rio Janeiro, Brazil). All other chemicals were obtained from Riedel-de-Haën (Charlotte, NC, USA).

### 2.2. Synthesis of Engineered Nanomaterials

Mesoporous silica nanocontainers (SiNCs) were synthesized according to Chen and collaborators [41] and the encapsulation of SEA-NINE™ 211N in SiNC (SiNC-DCOIT) is described in Maia et al. [8]. The nanomaterial synthesis is detailed in the Supplementary Material. Briefly, the formation of silica nanocapsules and the biocide encapsulation occurs in one step, resulting from an oil-in-water microemulsion polymerization process. Mesoporous capsules with differentiated porosity from core to outer shell regions were generated due to the gasification of solvents (oil phase) arising from the exothermic polymerization of TEOS.

Both ENMs were characterized previously regarding textural properties (Table S1), biocide loading and release [8]. Additionally, the chemical structure of the antifouling nanomaterial was confirmed based on Fourier-transform infrared spectroscopy (FTIR) spectra and the biocide degradation profile in seawater obtained via high-performance liquid chromatography (HPLC) [26].

### 2.3. Test Solutions and Dispersions

SEA-NINE™ 211N (hereinafter referred as DCOIT) was first dried for 30 min under 140 °C in a dry oven to evaporate xylene. The same drying step was performed in SiNC and SiNC-DCOIT. The test dispersions were prepared (1 mg/L, free or as SiNC-DCOIT) in the following: (i) low ionic strength medium, ultrapure water (UP water, Milli-Q water 18.2 MΩ, 25 °C); and (ii) two high ionic strength media, 0.45 μm filtered artificial seawater with 35 salinity (ASW, detailed composition in Table S2) and f/2-enriched [42] in filtered ASW with 35 salinity (f/2 medium, detailed composition in Table S3). The dispersions were placed in an ultrasonic water bath (Selecta; 550 W; 40 kHz, 25 °C) for 30 min.

### 2.4. Engineered Nanomaterial Characterization

ENM size and morphology were characterized via scanning electron microscopy (SEM) (Hitachi SU-70; Tokyo, Japan) coupled with energy dispersive spectroscopy using an electron beam energy of 15 kV. Both nanomaterial suspensions were prepared in UP water (1 mg/L), and the corresponding external particle diameter was determined using ImageJ (NIH, Bethesda, MD, USA).

Intensity-based dynamic light scattering (hydrodynamic size, $\overset{-}{x}DLS$) and surface charge (zeta potential, ζP) measurements were carried out on a Zetasizer Nano-ZS (Malvern Panalytical, UK) in 0.01, 0.5 and 1.0 mg/L of SiNC and SiNC-DCOIT.

The samples were initially pre-treated with an ultrasonic water-bath during 30 min prior to the analysis and afterwards stored in ambient conditions, in a closed vessel and protected from light.

The hydrodynamic size was monitored at times 0, 24 and 48 h, using polystyrene cuvettes (DTS0012, Malvern Panalytical), default high sensitivity settings of 173° backscatter detection, and the hydrodynamic diameter calculated using the Strokes-Einstein equation. As previously described, the ζP was determined in suspensions of ENMs in UP water using a capillary cuvette (DTS1060, Malvern Panalytical) and the Smoluchowski’s equation was used to derive the ζP. Except for SEM analysis, all the measurements were performed at 25 °C in triplicate.

### 2.5. Statistical Analysis

Shapiro-Wilk and Levene’s tests were performed to analyse the dataset normality and homoscedasticity, respectively (*p* = 0.05). Statistical differences in the ζP and $\overset{-}{x}$_DLS_ of each ENM, regarding different concentrations and time points, were established using a two-way ANOVA followed by a Holm-Šidák multiple comparison test whenever significant differences were attained (*p* < 0.05). The same approach was adopted to assess the effects of ionic strength on the $\overset{-}{x}$_DLS_ of each ENM. The statistical analyses were performed using SigmaPlot v.12.5 (Systat Software Inc., San Jose, CA, USA), and the results were expressed as average values (mean ± standard deviation). The variation within each parameter measured over time was assessed to estimate the stability of the colloidal suspensions [43].

## 3. Results and Discussion

### 3.1. Engineered Nanomaterial Morphology

**Figure 1:**
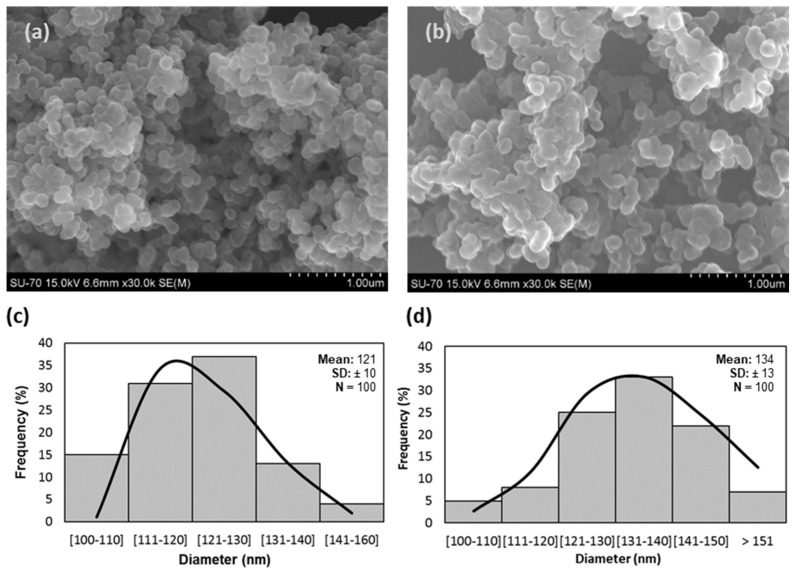
Scanning electron microscopy of (**a**) hollow silica nanocontainers (SiNC) and (**b**) SiNC with DCOIT encapsulated (SiNC-DCOIT), dispersed in ultra-pure water (1 mg/L). Histograms with the size distribution of (**c**) SiNC and (**d**) SiNC-DCOIT, determined with Figi package, ImageJ2 software.

SEM micrographs (Figure 1) showed spherical particles for both (a) SiNC and (b) SiNC-DCOIT with a mean external diameter (d) of 121 nm and 134 nm, respectively. The one-step SiNC synthesis produced homogenous nanocapsules with dimensions similar to what was reported in previous studies [8,9,26] (detailed in Table S1). According to the ISO 26824 [44], these ENMs fulfil the requirements to be considered a nanomaterial, as previous works demonstrate that internal pores were within the nanoscale [11].

The frequency histograms (Figure 1) with the particle size distribution of (c) SiNC and (d) SiNC-DCOIT revealed the prevalence of smaller particles in empty ENMs when compared to loaded nanocapsules; 50% of the particles were under 120 nm in SiNC, while more than 50% were larger than 130 nm in SiNC-DCOIT, which are related to the presence of large molecules of DCOIT.

The defined size of mesoporous silica, together with the successful encapsulation of organic molecules, makes this synthesis procedure a promising way of manufacturing antifouling nanomaterials for industrial applications [8,9], as it accelerates the production of these ENMs compared to other methods involving functionalization processes [10,45].

### 3.2. The Zeta Potential of ENMs

The lack of stability of colloidal ENMs can be considered a drawback while characterizing the exposure and ENM behaviour within an environmental risk assessment procedure, as it decreases reproducibility between studies and compromises data analysis [43,46,47].

The ζP of both ENMs was assessed over time to evaluate the stability of the suspensions in UP water. The results (Table 1 and Figure S3) showed that both ENMs were initially below the threshold for charge-stabilized nanoparticles (±30 mV), indicating colloidal instability for synthesized ENMs.

Previous studies testing hollow SiNC and SiNC loaded with organic molecules reported similar ζP values under this pH range, corroborating our results [10,12,26,48]. Conversely, other authors reported positive ζP values when cationic surfactants remain encapsulated [11,49], thus highlighting the role of loaded molecules in the overall nanocontainer surface charge. The ζP values increased over time for SiNC (*p* ≤ 0.01), reaching the −30 mV threshold after 48 h, but remained approximately −15 mV for SiNC-DCOIT until the end of the essay.

Based on the stability criteria defined by the OECD GD317 (2021) which set ±20% as the maximum deviation from the initial value, the dispersion of SiNC-DCOIT is considered stable, but not the SiNC. Ambrosone and co-authors (2014) [50] justify the silica reactivity with the presence of surface silanol groups, which can explain the results obtained for SiNC. Concerning SiNC-DCOIT, the same silanol groups could react with functional groups from DCOIT molecules, thus reducing the availability of the former groups to be solvated and contributing to the overall surface charge. However, previous studies that described the release of DCOIT from these silica nanocapsules [8] revealed that DCOIT could be almost fully extracted using organic solvents, implying that the interaction between DCOIT and silanol groups is not irreversible. Furthermore, other reasons that may explain differences in the surface charge of empty (SiNC) and DCOIT-loaded (SiNC-DCOIT) nanocapsules include differences in the extent of the TEOS reaction and degree of surfactant impurities staying in the nanocapsules after synthesis.

There is evidence showing that SiNC colloidal stability could be improved by adjusting the synthesis conditions, such as the catalyst concentration [51] or the biocide concentration [15].

Understanding the ENM stability is important while studying their effects in biota. The presence of organisms, leading to the release of excretion material, organic matter or other potential ligands, is a key factor affecting stability measurements [31,52,53]. Therefore, the OECD GD317 advises the use of a flowchart regarding test media manipulation, where natural organic matter (NOM) is suggested in specific cases as a nanomaterial’s stabilizer [43]. This is generally stated in freshwater systems [53] and is likely to occur in saline waters [54,55,56,57].

Herein, NOM was absent from the test media. However, studies demonstrated that NOM present in seawater has a minor effect on the stabilization of some ENMs, delaying but not preventing nanoparticle sedimentation [52,57,58]. According to the classical Derjaguin, Landau, Verwey and Overbeek (DVLO) theory [59], colloidal particles are surrounded by a diffuse electrical double layer (EDL) and the balance between the attractive (e.g., Van der Walls) and repulsive (e.g., EDL) forces determines the colloidal stability and, consequently, the state of aggregation of nanoparticles. High-electrolyte solutions, such as seawater, contribute to a reduction in the thickness of the EDL that surrounds nanoparticles, thus reducing the repulsive electrostatic interactions that modulate particle dispersion [60]. This electrostatic destabilization explains why the steric repulsion effect induced by different types of NOM [52,61] may not prevent particle aggregation in seawater. For instance, the presence of increasing concentrations of divalent cations in NOM-coated metal nanomaterials reduced the overall energy barriers between particles and was associated with increases in particle size [60].

Thus, we speculate that the low NOM present in seawater [56] will not prevent the aggregation and the gravitation settling of synthesized ENMs, although more studies are required.

### 3.3. The Influence of Ionic Strength on the ENM Hydrodynamic Diameter

Colloidal stability determines the state of aggregation of ENMs [60] and is modulated, among other factors, by the electrolyte composition and the ionic strength of surrounding media [22,27,56,61]. This study assessed the $\overset{-}{x}$DLS of produced ENMs when suspended in media of different ionic strengths and electrolyte compositions: (i) low ionic strength (UP water) and (ii) high ionic strength (ASW and f/2 medium) (Table 2 and Tables S4 and S5).

The DLS analysis recorded values of the polydispersity index above 0.7, reinforcing the heterogeneous nature of the dispersions formed. Consequently, Z-average estimations do not fulfil the set quality criteria. Thus, the ENM’s hydrodynamic size was based on the average value of peaks obtained in size distribution histograms (intensity-based) on the instrument software (Figures S1 and S2).

Initially (0 h), the increase in the ionic strength (UP < ASW < f/2) promoted the agglomeration of SiNC, with the largest complexes being detected in f/2 medium at concentrations 0.01 mg/L ($\overset{-}{x}$DLS = 365 ± 43.9 nm) and 0.5 mg/L ($\overset{-}{x}$DLS = 374 ± 55.5 nm), compared to the values obtained in UP water ($\overset{-}{x}$DLS = 148 ± 11.0 nm and $\overset{-}{x}$DLS = 103 ± 44.4 nm for 0.01 and 0.5 mg/L, correspondingly; *p* < 0.05). Similar results were detected at 0.01 mg/L SiNC-DCOIT ($\overset{-}{x}$DLS = 169 ± 43.9; 233 ± 43.9; 196 ± 94.8 nm for UP water, ASW and f/2 medium, respectively), but no statistical differences were noted at the highest concentrations. It is hypothesized that the mono (Na^+^, Cl^−^) and divalent ions (Mg^2+^; SO^2−^) in the ASW (Table S2) might adsorb to the surface of ENMs, causing electrostatic destabilization and, consequently, promoting particle agglomeration as described by other authors [22,23,27,35]. In the f/2 medium, ferric chloride contributed to the formation of larger particle complexes, as trivalent cations are known to exert a high electrostatic destabilization effect on nanoparticles [62].

For the highest tested concentration (1.0 mg/L), differences in the media ionic strength and composition had no significant effect on the ENMs’ hydrodynamic size. However, particle enlargement and bimodal size distribution were evident, suggesting the presence of agglomerates. When the concentration increases, the frequency of collisions between particles is enhanced, which facilitates the agglomeration of ENMs [63,64]. Our data suggest that the particle concentration may mask the effects of ionic strength on the agglomeration state of ENMs above a certain concentration threshold. Therefore, defining an optimal concentration range to obtain monodispersed ENMs should be prioritized when evaluating the stability of nanoparticles in different aqueous dispersions [65].

Time promoted the formation of agglomerates of larger dimensions for both ENMs in the conditions tested, but the effects were more evident with low ionic strength media. In UP water, particle complexes with average dimensions of 451, 318 and 264 nm were reported at 48 h for 0.01, 0.5 and 1.0 mg/L SiNC, respectively. Meanwhile, in ionic-rich media, the minimum and maximum hydrodynamic sizes detected were 235–287 nm for ASW and 276–363 nm for f/2 medium. In SiNC-DCOIT, unimodal dispersions and particle enlargement were evident in UP water by the end of the assay (209, 212 and 237 nm for 0.01, 0.5 and 1.0 mg/L SiNC, respectively), but differences between time points were only significant at 1.0 mg/L in f/2 medium.

Because particle enlargement occurred along with variations on the ζP value for SiNC, as described in Section 3.2, it is hypothesized that aggregation promoted the electrostatic stabilization of SiNC by reducing the surface energy, as reported for other ENMs [51,64]. The same was not verified for SiNC-DCOIT, highlighting that biocide chemistry modulates nanomaterial surface reactivity and the agglomeration state.

A decrease in the particle size was registered in 1.0 mg/L SiNC suspended in UP water after 48 h, suggesting the removal of SiNC from the water column by settling, while in the f/2 medium, the same was recorded after 24 h in 1.0 mg/L SiNC. These results indicate faster deposition of ENM aggregates in seawater and differ from what was reported for SiO_2_ NPs, which can last for months in the water column [39]. In SiNC-DCOIT, the average particle size was reduced from 24 h to 48 h in 0.5 and 1.0 mg/L in UP water and in 1.0 mg/L in the f/2 medium. Information on the sedimentation time of ENMs is critical to predicting shelf time and the potential environmental sink and impacts of those ENMs.

## 4. Conclusions

The agglomeration pattern observed in SiNC (widely used in nanotechnology) and SiNC-DCOIT (the novel antifouling nanomaterial) indicates a lack of colloidal stability of manufactured nanomaterials in both low- and high-ionic-strength media, as confirmed by the zeta potential and hydrodynamic size estimations. The encapsulation of DCOIT biocide in SiNC contributed to stabilizing the nanomaterial when dispersed. However, the extent of electrostatic repulsive forces was insufficient to prevent particle agglomeration.

Saline media, either a natural matrix or presenting as a defined composition, are characterized by high ionic content and complexity. Given the lack of specific nanosafety guidelines for marine water quality, this dataset is paramount to interpreting ecotoxicological results more reliably, as confirmed by the agglomeration or aggregation of nanoparticles and, eventually, sedimentation occurring in high-ionic media.

The present results highlight the aggregation of these novel ENMs and the increased bioavailability of these nanomaterials to sessile and sediment-dwelling marine organisms. Despite the benefits of reduced toxicity described for SiNC-DCOIT compared to free DCOIT, the risks associated with the release of coating pain particles containing ENMs should not be neglected.

## Figures and Tables

**Table 1:** Zeta potential values (ζP, mV) in suspensions of hollow silica nanocontainers (SiNC: 0.01, 0.5 and 1.0 mg/L) or with encapsulated DCOIT (SiNC-DCOIT: 0.01, 0.5 and 1.0 mg DCOIT/L), dispersed in ultra-pure water at 0, 24 and 48 h post resuspension. Data are expressed as averaged values ± SD (*n* = 3). Superscript letters ^(a, b)^ indicate homogeneous groups within each nanomaterial (SiNC or SiNC-DCOIT) and tested concentration, over time (*p* < 0.05). Symbols ^(^*^, #, $)^ denotes homogeneous groups in each nanomaterial (SiNC or SiNC-DCOIT) and timepoint, due to concentration variation (*p* < 0.05).

Nanomaterial	Concentration (mg/L)	Time (h)	pH	ζP (Mean ± SD, mV)	
SiNC	0.01	0	6.4	−8.7 ± 0.2	^(a,^*^)^
		24	6.6	−28.3 ± 3.0	^(b)^
		48	6.7	−23.9 ± 2.0	^(b,^*^)^
	0.5	0	6.8	−11.6 ± 0.6	^(a,^*^#)^
		24	6.7	−31.8 ± 3.7	^(b)^
		48	6.7	−32.6 ± 2.4	^(b,#)^
	1.0	0	6.6	−16.6 ± 1.5	^(a,#)^
		24	6.7	−31.7 ± 2.5	^(b)^
		48	6.8	−29.8 ± 5.5	^(b,#)^
SiNC-DCOIT	0.01	0	6.2	−12.2 ± 0.6	^(a,^*^)^
		24	6.4	−16.3 ± 1.5	^(b)^
		48	6.7	−15.1 ± 1.1	^(b,^*^#)^
	0.5	0	6.0	−15.3 ± 0.9	^(b,#)^
		24	6.0	−14.3 ± 1.7	^(ab)^
		48	6.3	−13.2 ± 0.4	^(a,^*^)^
	1.0	0	6.2	−17.1 ± 0.1	^($)^
		24	6.2	−16 ± 0.8	
		48	6.4	−15.4 ± 0.3	^(#)^

**Table 2:** Hydrodynamic size (nm) of hollow silica nanocontainers (SiNC: 0.01, 0.5 and 1.0 mg/L) or with encapsulated DCOIT (SiNC-DCOIT 0.01, 0.5 and 1.0 mg/L) in ultrapure water (UP), artificial seawater (ASW) or f/2-enriched seawater medium (f/2 medium) at 0, 24 and 48 h post-resuspension. Data are expressed as averaged values ± SD (*n* = 3). Superscript letters ^(a, b)^ indicate statistical differences (*p* < 0.05) between test media, within each combination of concentration and time, for each nanomaterial (SiNC or SiNC-DCOIT). Symbols (*^, #, $^) denote significant differences (*p* < 0.05) over time, within each concentration and test media, for each nanomaterial.

Nanomaterial	Concentration (mg/L)	Time (h)	Size (nm)					
			UP		ASW		f/2 Medium	
SiNC	0.01	0	148 ± 11.0	^(a,^*^)^	161 ± 36.6	^(a,^*^)^	365 ± 43.9	^(b,#)^
		24	272 ± 51.2	^(#)^	269 ± 70.7	^(#)^	279 ± 27.4	^(^*^)^
		48	451 ± 40.1	^(b,$)^	262 ± 51.9	^(a,#)^	276 ± 21.6	^(a,^ * ^)^
	0.5	0	103 ± 44.4	^(a,^*^)^	257 ± 103.2	^(ab)^	374 ± 55.5	^(b)^
		24	320 ± 112.0	^(#)^	223 ± 22.5		312 ± 30.2	
		48	318 ± 119.3	^(#)^	287 ± 12.9		310 ± 60.5	
	1.0	0	235 ± 113.7		176 ± 122.6		336 ± 47.0	
		24	456 ± 203.6		224 ± 57.7		334 ± 14.5	
		48	264 ± 63.8		235 ± 29.9		363 ± 13.8	
SiNC-DCOIT	0.01	0	169 ± 53.5	^(a,^*^)^	233 ± 26.0	^(b)^	196 ± 94.8	^(b)^
		24	262 ± 18.6	^(b,#)^	200 ± 25.4	^(a)^	190 ± 5.5	^(a)^
		48	209 ± 15.6	^(ab,^*^#)^	266 ± 9.1	^(b)^	203 ± 45.2	^(a)^
	0.5	0	194 ± 6.1		195 ± 56.6		180 ± 32.2	
		24	246 ± 29.5		201 ± 3.6		199 ± 58.8	
		48	212 ± 9.1		192 ± 55.1		169 ± 24.6	
	1.0	0	152 ± 20.3	^(^*^)^	189 ± 69.0		254 ± 90.2	^(^*^)^
		24	241 ± 23.5	^(#)^	220 ± 69.6		344 ± 61.0	^(#)^
		48	237 ± 43.2	^(#)^	240 ± 104.0		310 ± 84.6	^(^*^#)^

## Data Availability

The data presented in this study are available on request from the corresponding author.

## Supplementary Materials

The following supporting information can be downloaded at: https://www.mdpi.com/article/10.3390/nano13111738/s1. Figure S1: Histograms with particle size distribution (nm) based on the dynamic light scattering properties of hollow silica nanocontainers (SiNC) in ultra-pure water at distinct concentrations (0.01, 0.5 and 1.0 mg/L of SiNC) and timepoints (0, 24, 48 h); Figure S2: Histograms with particle size distribution (nm) based on the dynamic light scattering properties of DCOIT encapsulated in silica nanocontainers (SiNC-DCOIT) in ultra-pure water at distinct concentrations (0.01, 0.5 and 1.0 mg/L of SiNC) and timepoints (0, 24, 48 h); Figure S3: Zeta potential (ζP, mV) in suspensions of 0.01, 0.5 and 1.0 mg/L of (a) hollow silica nanocontainers (SiNC) or (b) with encapsulated DCOIT (SiNC-DCOIT), dispersed in ultra-pure water, at 0, 24 and 48 h. Data is expressed as averaged values ± SD (*n* = 3); Table S1: Textural properties of hollow silica nanocontainers (SiNCs) and DCOIT encapsulated in silica nanocontainers (SiNC-DCOITs); Table S2: Chemical composition of artificial seawater, 35 salinity; Table S3: ASW enrich f/2 media recipe, dissolving the list of inorganic reagents in ASW; Table S4: Analysis of Variance (Two-way ANOVA, main effects) testing for effects due to time (0, 24, 48 h) and test media (ultrapure water (UP); artificial seawater (ASW) or f/2-enrich in ASW (f/2)) on the values of hydrodynamic size to hollow silica nanocontainers (SiNC). Significant differences (*p* = 0.05) are indicated in bold. Abbreviations: sum-of-squares (SS), degrees of freedom (*df*), mean squares (MS), the F ratio (*F*) and the P value (*P*); Table S5: Analysis of Variance (Two-way ANOVA) testing for effects due to time (0, 24, 48 h) and test media (ultrapure water (UP); artificial seawater (ASW) or f/2-enrich in ASW (f/2)) on the hydrodynamic size to DCOIT containing silica nanocontainers (SiNC-DCOIT). Significant differences (*p* = 0.05) are indicated in bold. Abbreviations: sum-of-squares (SS). degrees of freedom (*df*). mean squares (MS). the F ratio (*F*) and the P value (*P*). References [66,67] are cited in the Supplementary Materials.
