# Supplementary material for: Characterization and Behaviour of Silica Engineered Nanocontainers in Low and High Ionic Strength Media
Source: Nanomaterials (Basel). 2023 May 26;13(11):1738. doi: 10.3390/nano13111738 (PMC10254789; doi:10.3390/nano13111738)
Supplement: Supplementary file 1 [file nanomaterials-13-01738-s001.zip › nanomaterials-2401964-supplementary.pdf]

Supplementary material

Experimental

Synthesis of engineered nanomaterials

The synthesis of mesoporous silica nanocontainers (SiNC) was performed based on Chen and collaborators (2008) and for generating the antifouling nanomaterial (SiNC-DCOIT), adaptations to the previous procedure were made according to Maia et al. (2015).

For the chemical reactions, cetyltrimethylammonium bromide (CTAB) was used as cationic surfactant and porogen agent, ammonium hydroxide (25% v/v) was used as catalyst and two different cosolvent mixtures were added: a) ethyl ether (for SiNC); or b) a mixture of ethyl ether:xylene (4:1) plus DCOIT (for SiNC-DCOIT).

Initially, CTAB (0.71% (w/v) in water) was mixture to the ammonia solution (0.25 mL). Then, ethyl ether or a mixture of ethyl ether:xylene (4:1) plus DCOIT were added for generating SiNC or SiNC-DCOIT, correspondingly, and an oil-in-water microemulsion was obtained. The solutions were left to stabilize for 30 min and 2 mL of TEOS was subsequently added dropwise to the emulsion. The synthesis reaction occurred during 24 h at room temperature, under constant stirring. The resulting nanomaterials (SiNC and SiNC-DCOIT) were then filtered, washed and dried at room temperature. Both nanomaterials were additionally dried for 30 min under 140 °C in a dry oven to allow for xylene residues evaporation and stored at room temperature, in a dry placed, protected from light.

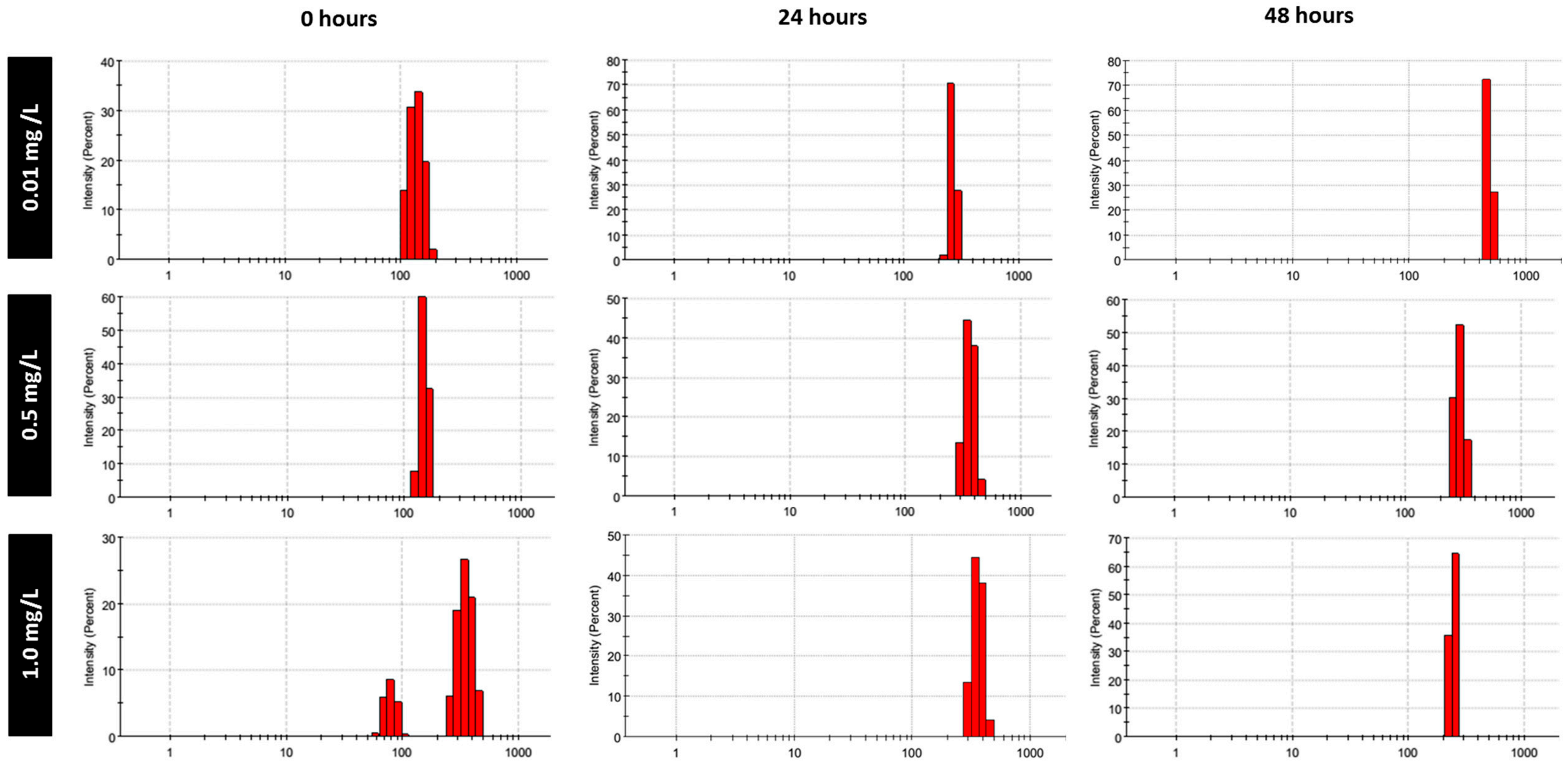

Figure S1: Histograms with particle size distribution (nm) based on the dynamic light scattering properties of hollow silica nanocontainers (SiNC) in ultra-pure water at distinct concentrations (0.01, 0.5 and 1.0 mg/L of SiNC) and timepoints (0, 24, 48 h).

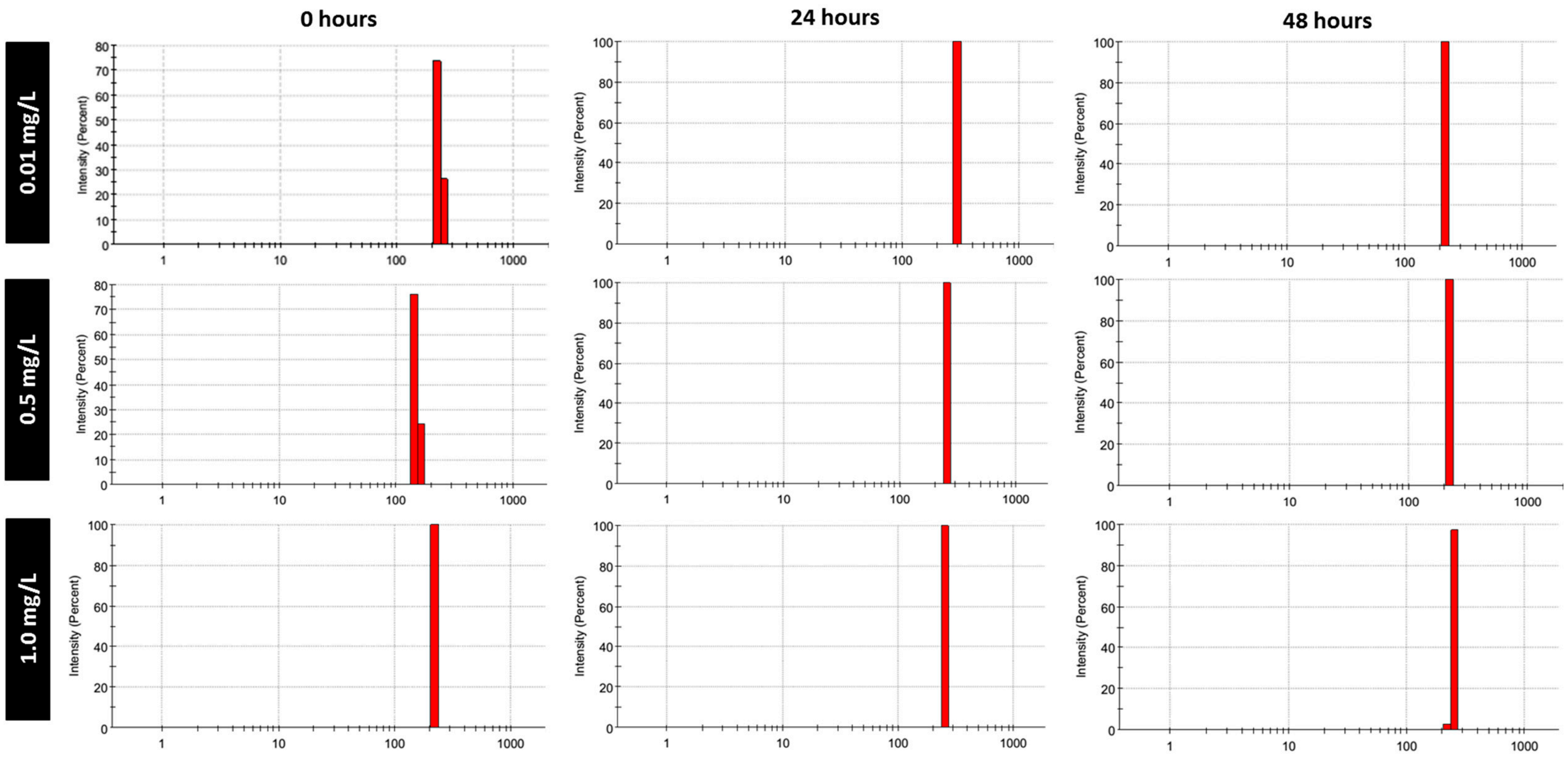

Figure S2: Histograms with particle size distribution (nm) based on the dynamic light scattering properties of DCOIT encapsulated in silica nanocontainers (SiNC-DCOIT) in ultra-pure water at distinct concentrations (0.01, 0.5 and 1.0 mg/L of SiNC) and timepoints (0, 24, 48 h).

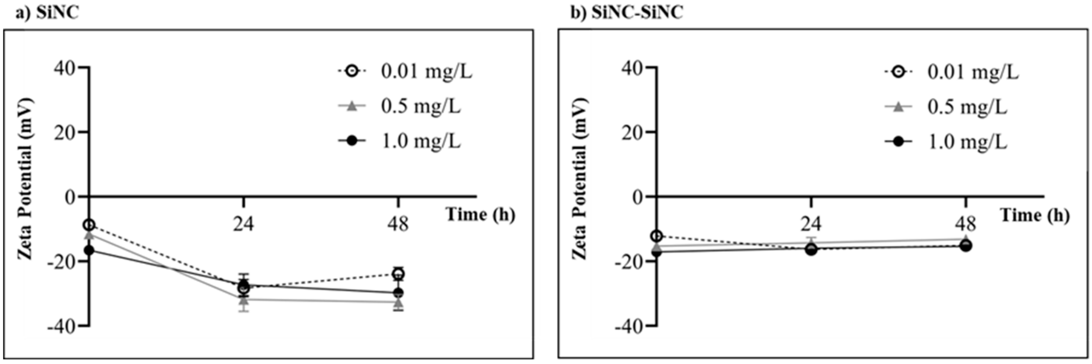

Figure S3. Zeta potential (ζP, mV) in suspensions of 0.01, 0.5 and 1.0 mg/L of (a) hollow silica nanocontainers (SiNC) or (b) with encapsulated DCOIT (SiNC-DCOIT), dispersed in ultra-pure water, at 0, 24 and 48 h. Data is expressed as averaged values ± SD (n = 3).

Table S1: Textural properties of hollow silica nanocontainers (SiNC) and DCOIT encapsulated in silica nanocontainers (SiNC-DCOIT) determined from the nitrogen adsorption-desorption isotherms (adapted from Maia et al., 2015). Abbreviations: S_{BET}: surface area, BET (Brunauer–Emmett–Teller) method (Brunauer et al., 1938); Φpore: pore diameter, BJH (Barrett–Joyner–Halenda) method (Gregg et al., 1967).

Nanomaterial	S_{BET}/m^2 g^{-1}	Φ_{pore} BJH[a]/nm	Φ_{pore} BJH/nm (maximum)
SiNC	157	2.7	37
SiNC-DCOIT	214	2.7	50

Table S2: Macronutrients content in artificial seawater (ASW), 35 salinity.

Specification	Concentration	
	(g/L)	(mM)
Chlorine (Cl^-)	17.15	484
Sodium (Na^+)	9.80	426
Magnesium (Mg^{2+})	1.44	59
Sulfide (S^{2-})	0.77	24
Calcium (Ca^{2+})	0.49	12
Potassium (K^+)	0.38	10

Table S3: ASW enrich f/2 media recipe, dissolving the list of inorganic reagents in ASW.

Specification	Concentration	
	(mg/L)	(nM)
Sodium nitrate ($NaNO_3$)	75	882.46
Monosodium dihydrogen ortophosphate (NaH_2PO_4)	5	36.24
Trace metals		
EDTA dissoldium salt ($Na_2EDTA . 2H_2O$)	4.40	11.76
Ferric Chloride hexahydrate ($FeCl_3 . 6H_2O$)	3.20	11.84
Manganese(II) chloride tetrahydrate ($MnCl_2 . 4 H_2O$)	0.179	0.91
Zinc sulfate heptahydrate ($ZnSO_4 . 7H_2O$)	0.022	0.08
Cobalt(II) chloride ($CoCl_2 . 6H_2O$)	0.010	0.04
Cupric sulfate ($CuSO_4 . 5H_20$)	0.010	0.04
Molybdic acid sodium salt dihydrate ($Na_2MoO_4 . 2H_20$)	0.006	0.03

Table S4: Analysis of Variance (Two-way ANOVA, main effects) testing for effects due to time (0, 24, 48h) and test media (ultrapure water (UP); artificial seawater (ASW) or f/2-enrich in ASW (f/2)) on the values of hydrodynamic size to hollow silica nanocontainers (SiNC). Significant differences (p = 0.05) are indicated in bold. Abbreviations: sum-of-squares (SS), degrees of freedom (*df*), mean squares (MS), the F ratio (*F*) and the P value (*P*).

**Hydrodynamic diameter**					
Concentration: 0.01 mg/L					
**Source**	***df***	***SS***	**MS**	***F***	***P***
Timepoint	2	44481.1	22240.5	14.1	**<0.001**
Test media	2	25125.2	12562.7	8.0	0.003
Timepoint x Test media	4	136889.4	34222.3	21.7	**<0.001**
Residual	18	28408.4	1578.2		
Total	26	234904.1	9034.8		
Concentration: 0.5 mg/L					
**Source**	***df***	***SS***	**MS**	***F***	***P***
Timepoint	2	15849.526	7924.8	1.1	0.341
Test media	2	2417.412	1208.7	0.2	0.841
Timepoint x Test media	4	133060.670	33265.2	4.8	**0.008**
Residual	18	124653.933	6925.2		
Total	26	275981.542	10614.7		
Concentration: 1.0 mg/L					
**Source**	***df***	***SS***	**MS**	***F***	***P***
Timepoint	2	51815.878	25907.9	2.9	0.079
Test media	2	39829.133	19914.5	2.2	0.134
Timepoint x Test media	4	58213.348	14553.3	1.6	0.206
Residual	18	158867.270	8826.0		
Total	26	308725.630	11874.0		

Table S5: Analysis of Variance (Two-way ANOVA) testing for effects due to time (0, 24, 48h) and test media (ultrapure water (UP); artificial seawater (ASW) or f/2-enrich in ASW (f/2)) on the hydrodynamic size to DCOIT containing silica nanocontainers (SiNC-DCOIT). Significant differences (*p* = 0.05) are indicated in bold. Abbreviations: sum-of-squares (SS). degrees of freedom (*df*). mean squares (MS). the F ratio (*F*) and the P value (*P*).

**Hydrodynamic diameter**					
Concentration: 0.01 mg/L					
**Source**	***SS***	***df***	**MS**	***F***	***P***
Timepoint	2	905.9	453.0	0.5	0.594
Test media	2	3020.0	1510.0	1.8	0.196
Timepoint x Test media	4	21634.1	5408.5	6.4	**0.002**
Residual	18	15221.1	845.6		
Total	26	40781.1	1568.5		
Concentration: 0.5 mg/L					
**Source**	***SS***	***df***	**MS**	***F***	***P***
Timepoint	2	6161.3	3080.6	1.0	0.392
Test media	2	4161.6	2080.8	0.7	0.526
Timepoint x Test media	4	6375.5	1593.9	0.5	0.729
Residual	18	56177.1	3120.9		
Total	26	72875.6	2802.9		
Concentration: 1.0 mg/L					
**Source**	***SS***	***df***	**MS**	***F***	***P***
Timepoint	2	45164.4	22582.2	5.0	**0.019**
Test media	2	113870.0	56934.9	12.6	**<0.001**
Timepoint x Test media	4	22126.1	5531.5	1.2	0.336
Residual	18	81430.5	4523.9		
Total	26	262590.8	10099.6		

References

Brunauer, S., Emmett, P.H., Teller, E., 1938. Adsorption of Gases in Multimolecular Layers. J Am Chem Soc 60, 309–319. https://doi.org/10.1021/ja01269a023

Chen, H., He, J., Tang, H., Yan, C., 2008. Porous Silica Nanocapsules and Nanospheres: Dynamic Self-Assembly Synthesis and Application in Controlled Release. Chemistry of Materials 20, 5894–5900. https://doi.org/10.1021/cm801411y

Gregg, S.J., Sing, K.S.W., Salzberg, H.W., 1967. Adsorption Surface Area and Porosity. J Electrochem Soc 114, 279C. https://doi.org/10.1149/1.2426447

Maia, F., Silva, A.P., Fernandes, S., Cunha, A., Almeida, A., Tedim, J., Zheludkevich, M.L., Ferreira, M.G.S., 2015. Incorporation of biocides in nanocapsules for protective coatings used in maritime applications. Chemical Engineering Journal 270, 150–157. https://doi.org/10.1016/j.cej.2015.01.076
